# Prevalence, causes and risk factors of vision impairment in states of Andhra Pradesh and Telangana in South India: the Longitudinal Eye health, Ageing and Disability Study

**DOI:** 10.1136/bmjophth-2026-003146

**Published:** 2026-11-06

**Authors:** Srinivas Marmamula, Vidya Paramparambath, Kavitha Kummari, Charulatha Sekar, Thirupathi Reddy Kumbham, Venkateswara Rao Pilla, Swetha Krishnan, Abhinaya Mohan, Joshua R Ehrlich, David E Bloom

**Affiliations:** 1 Wellcome Trust/Department of Biotechnology India Alliance, L V Prasad Eye Institute, Hyderabad, Telangana, India; 2 Allen Foster Community Eye Health Research Centre, Gullapalli Pratibha Rao International Centre for Advancement of Rural Eye care, L V Prasad Eye Institute, Hyderabad, Telangana, India; 3 Brien Holden Institute of Optometry and Vision Sciences, Prof. Brien Holden Eye Research Centre, L V Prasad Eye Institute, Hyderabad, India; 4 Ophthalmology and Visual Sciences, University of Michigan, Ann Arbor, Michigan, USA; 5 University of Michigan Institute for Social Research, Ann Arbor, Michigan, USA; 6 Department of Global Health and Population, Harvard T H Chan School of Public Health, Boston, Massachusetts, USA

**Keywords:** Vision, Public health, Prospective Studies

## Abstract

**Objective:**

To report the prevalence, causes and risk factors for vision impairment (VI) among individuals aged ≥60 years in Andhra Pradesh and Telangana in South India.

**Methods:**

We conducted a population-based cross-sectional study in 120 randomly selected clusters across urban and rural regions. Makeshift clinics were established and trained teams performed detailed eye examinations, including visual acuity assessment for distance and near. In-depth interviews were conducted to gather personal, demographic and other health-related information. VI was defined as presenting visual acuity worse than 6/12 in the better eye. A multiple logistic regression analysis was performed to assess factors associated with VI.

**Findings:**

Of 7200 individuals enumerated, 6291 (87.4%) were examined. The mean (±SD) age of those examined was 68.9 (±7.3) years and 54.46% (n=3436) were women. The age-adjusted and gender-adjusted prevalence of VI was 42.56% (95% CI 40.52% to 44.63%). VI was associated with older age groups, lower levels of education, current or past alcohol consumption and current smoking. Non-working individuals and residents of the Rayalaseema region had higher odds of VI, whereas self-reported diabetes and hypertension were associated with lower odds. Uncorrected refractive errors (52.43%), cataract (34.75%) and posterior capsular opacification (6.41%) after cataract surgery were the leading causes of VI.

**Conclusions:**

Four out of every 10 individuals aged ≥60 years had VI. Over 85% of VI in this population can be addressed by providing spectacles and cataract surgery. Age-friendly eye care programmes that prioritise these two interventions must be strengthened to reduce VI in the region.

**WHAT IS ALREADY KNOWN ON THIS TOPIC**

- Vision impairment is common in the older population in India.

**WHAT THIS STUDY ADDS**

- Provides detailed insights into the burden, causes and risk factors of vision impairment from a large-scale population-based study specifically focused on older populations in two large states in South India.
- In addition, this study provides estimates of person-years lived with visual disability in the two states in India.

**HOW THIS STUDY MIGHT AFFECT RESEARCH, PRACTICE OR POLICY**

- Due to the increasing proportion of older people, the prevalence of vision impairment is expected to rise in the states of Andhra Pradesh and Telangana.
- As a large proportion of vision impairment is due to avoidable causes, cost-effective interventions specifically targeting high-risk groups are likely to help contain the rising number of older people with vision impairment, thereby contributing to healthy and active ageing in India.

## Introduction

 Vision impairment (VI) is a global public health concern affecting more than 1.1 billion people.^1^ VI is associated with older age; more than 70% of people with VI are 50 years or older. Cost effective interventions, such as spectacles or cataract surgery, can address more than 80% of VI.^2^ In individuals aged 60 and older, VI can lead to falls and accidents. These incidents may subsequently lead to fractures, negatively affecting their quality of life.^3–6^ VI is also associated with depression, and loss of functional independence and adversely impacts activities of daily living in older people.^7–11^ The number of people in the older age group is increasing; therefore, data on VI are required to plan eye care programmes.^12^ Addressing VI is crucial due to its intricate, far-reaching effects on other dimensions of health and well-being among older individuals. VI in older people is likely to affect their families and result in increased caregiver time, increased healthcare expenditure and dependency.^13^

Comprehensive eye health studies that focus exclusively on older people are uncommon in India. Several rapid assessment studies conducted in India include only basic eye examinations, are restricted to VI alone and include only a limited number of risk factors related to VI and other dimensions of health and ageing.^14^ Hence, comprehensive data on VI and risk factors are required for planning holistic healthcare interventions, particularly for older people. The earlier Hyderabad Ocular Morbidity in Elderly Study (HOMES) highlighted the burden of VI in older individuals living in care facilities.^15–17^ As HOMES included only individuals in residential care and was restricted to urban areas, the results cannot be extrapolated to older people in the communities.

The population-based Longitudinal Eye health, Ageing and Disability Study (LEADS) aims to bridge the knowledge gap and provide vital information on the status of eye health, including VI and its associations with systemic conditions and disabilities. The methodology used in the study has been reported in previous publication.^18^ This paper reports on the prevalence, causes and risk factors of VI among older adults in the states of Andhra Pradesh and Telangana in South India. It also reports on the personal and demographic characteristics of the LEADS participants.

## Materials and methods

### Study locations and sample size

LEADS was conducted among people aged ≥60 years in the states of Andhra Pradesh (comprising coastal Andhra Pradesh and Rayalaseema region) and Telangana, which together constituted the former combined state of Andhra Pradesh. According to the 2011 census, the total population of these regions is approximately 84 million. The study included seven districts across three regions: Guntur and Bapatla in coastal Andhra Pradesh; YSR Kadapa and Annamayya in Rayalaseema; and Adilabad, Mancherial and Kumuram Bheem Asifabad in Telangana. The total population of these seven districts is approximately 10 million.

Our previous publication describes the sample size estimation and sample selection process.^18^ In brief, the sample size was calculated using a conservative estimate of a 3% annual incidence of VI (defined as presenting visual acuity (VA) worse than 6/12 in the better eye) and a precision of 0.6% (20% of the prevalence).^19^ To account for a cluster size of 60 participants, a design effect of 1.6 was used. The sample size increased to account for a 30% non-response rate. The final sample size required was 7076, which was rounded up to 7200 across 120 clusters.

A two-stage cluster random sampling method was employed. Initially, clusters were systematically selected using probability proportional to size sampling from a sampling frame with all villages listed from the seven districts. Using the compact segment sampling technique, 60 participants aged ≥60 years were chosen in each cluster. This two-stage sampling method is mathematically self-weighting.^20^ In each segment, door-to-door visits were conducted, with consecutive enumeration of participants until the target of 60 participants was reached. Only individuals who fulfilled the age criteria and were residing in the chosen household for a minimum of 1 month before the enumeration date were included. Field investigators conducted detailed interviews of individuals who consented to participate in the study in their households. After the interviews, the participants were accompanied to the makeshift clinic set up in the vicinity on the same day or the next day, depending on their availability.

### Study teams

Trained clinical teams conducted eye examinations in makeshift clinics. Two multidisciplinary teams, each comprising optometrists, field investigators and vision technicians, collected the data. Domain experts provided rigorous, domain-specific training prior to the study to ensure standardisation. These teams were coordinated by the study coordinator and supported by data-entry personnel based at the central office in Hyderabad.

### Eye examination

A complete, comprehensive eye examination, including detailed ocular history and fundus imaging, was conducted as described in our previous publications.^16 18^ Visual Acuity (VA) for distance and near was assessed using a logarithm of the minimum angle of resolution (logMAR) chart with tumbling ‘E’ or English letter optotypes at three meters and 40 cm, respectively. Unaided VA was recorded in all cases. Aided VA was recorded if a participant was using spectacles. Refraction (manual and autorefraction) was performed for those with presenting distance VA worse than 6/9 or near vision worse than N6 in either eye. The best-corrected distance and near VA were measured and documented. The anterior segment was evaluated using a hand-held portable slit lamp (Remidio PSL camera, Remidio Innovative Solution Pvt., India). The eyelids, conjunctiva, cornea, anterior chamber, and lens were examined. Intraocular pressure was measured using a non-contact tonometer (iCare IC100, Finland). Fundus examination and retinal imaging were done using a non-mydriatic fundus camera (Remedio Fundus on Phone (FOP NM-10), Remidio Innovative Solutions Pvt. Ltd., India). Artificial intelligence grading algorithms were used for retinal imaging.^19 21^ A real-time tele-ophthalmology consultation with a specialist was arranged for acute conditions, if required. Spectacles were provided ‘on the spot’ after the eye examination or delivered directly to participants at their homes wherever required.

Individuals with mobility or other health issues were examined at home. All individuals with VI (other than refractive errors) or those who needed further consultation were referred to the nearest L V Prasad Eye Institute eye care centre. Participants received all services free of charge. Referrals to the appropriate specialists/primary health centres for systemic conditions were provided when required. Follow-up phone calls/visits were made to ensure compliance with the referral and spectacle use, and reasons for non-compliance were also assessed.

### Case definitions

VI is defined as presenting VA worse than <6/12 in the better eye, as defined by the WHO and used in India.^22 23^ VI was further subdivided into mild VI (worse than 6/12 to 6/18), moderate VI (worse than 6/18 to 6/60), severe VI (worse than 6/60 to 3/60) or blindness (worse than 3/60 to no light perception). VI due to cataract, uncorrected refractive errors (UREs) or posterior capsular opacification was defined as ‘avoidable’ VI. The main reason for VI was recorded individually for each eye and subsequently for the individual. In case of multiple causes, the one that is more easily treatable or correctable was documented as the primary cause of VI.^15^

### Independent variables

Other covariates comprised age groups (60–69, 70–79, ≥80 years), gender (male, female), education (no formal schooling, school education, higher education), mobility status (independent, bedridden/assisted living), employment (working, retired/never employed), smoking status (never, prior, current), alcohol consumption (never, prior, current), self-report of diabetes (yes/no) and hypertension (yes/no), location (urban, rural), and region (coastal Andhra Pradesh, Rayalaseema region, Telangana).

To assess the number of years lived with visual disability (YLVDs), the average duration of VI was considered to be 5 years as reported in a population-based study from the same region.^24^ Only those with moderate VI, severe VI and blindness were included to estimate YLVDs, assuming that mild VI may be a limited perceived disability in older people.

### Data management

Data were collected in the field using a tablet-based application, synced to the cloud and securely stored on an encrypted server. Stata Statistical Software for Windows, V.17, was used for the data analysis.^25^ To account for the two-stage cluster sampling design, variance estimation and 95% CIs were adjusted for clustering at the village level using Taylor series linearisation (svy commands in Stata). To account for differential response rates across age groups and gender, poststratification weights were applied and age-adjusted and gender-adjusted prevalences are presented with 95% CIs. Independent sociodemographic predictors of VI were evaluated using multivariable logistic regression and adjusted ORs are reported. Continuous variables are presented as means (SD), and categorical variables are presented as proportions. Between-group differences were assessed using t-test for continuous variables and χ² test for categorical variables. Statistical significance was set at p<0.05, but actual p values obtained were reported. In addition, the prevalence estimates are projected to the population of older people in the year 2050.

## Results

### Demographic characteristics of the participants

In total, 7200 individuals were enumerated, of which 6291 (87.4%) were examined from 120 clusters in the states of Andhra Pradesh (coastal region and Rayalaseema) and Telangana. The response rate was highest in Telangana (89.1%), followed by coastal Andhra Pradesh (87.2%) and the Rayalaseema region (85.8%). The response rates were similar among men and women (87.2% vs 87.5%; p=0.71). Those examined were older than those not examined (68.9 (±7.3) years vs 64.7 (±7.1) years; p<0.01).

Among those examined, 54.46% (n=3436) were women. In terms of education, 57.42% (n=3612) had no formal education, 39.99% (n=2516) had school education and 2.59% (n=163) had higher education. Overall, 74.2% (n=4668) were from rural areas; 42.55% (n=2677) and 21.67% (n=1363) reported hypertension and diabetes, respectively. About 10% (n=609) of participants were bedridden or had other health issues requiring home examination. About a quarter of the participants had a previous or current smoking history or a history of alcohol consumption. Nearly one-third of the participants from each of the three locations were examined: Coastal Andhra Pradesh (33.27%; n=2093), Rayalaseema (32.73%; n=2059), and Telangana (34.00%; n=2139).

### Prevalence of VI

Table 1 shows the categories of VI stratified by region. Overall, the age-adjusted and gender-adjusted prevalence of VI was 42.56% (95% CI 40.52% to 44.63%). The prevalence of VI varied significantly, with a high prevalence in the Rayalaseema region (47.57%; 95% CI 44.33% to 50.83%), followed by Telangana (43.31%; 95% CI 40.26% to 46.40%) and the lowest in coastal Andhra Pradesh (36.88%; 95% CI 33.55% to 40.34%) (table 1).

**Table 1:** Categories of VI in the states of Andhra Pradesh and Telangana

Presenting visual acuity in the better eye	Andhra Pradesh				Telangana (n=2139)		Total (n=6291)	
	Rayalaseema region (n=2059)		Coastal Andhra region (n=2093)					
	n	Prevalence (%) (95% CIs)	n	Prevalence (%) (95% CIs)	n	Prevalence (%) (95% CIs)	N	Prevalence (%) (95% CIs)
Mild VI (<6/12 to 6/18)	331	16.08 (14.51 to 17.73)	346	16.53 (14.96 to 18.19)	353	16.50 (14.95 to 18.14)	1030	16.37 (15.47 to 17.31)
Moderate VI (<6/18 to 6/60)	552	26.81 (24.90 to 28.77)	358	17.10 (15.51 to 18.78)	470	21.97 (20.23 to 23.79)	1380	21.94 (20.92 to 22.98)
Severe VI (<6/60 to 3/60)	42	2.04 (1.47 to 2.74)	45	2.15 (1.57 to 2.86)	61	2.85 (2.19 to 3.65)	148	2.35 (1.99 to 2.76)
Blindness (<3/60 to no light perception)	54	2.62 (1.98 to 3.41)	33	1.58 (1.09 to 2.21)	54	2.52 (1.90 to 3.28)	141	2.24 (1.89 to 2.64)
Crude prevalence of VI	979	47.55 (45.37 to 49.73)	782	37.36 (35.28 to 39.47)	938	43.85 (41.73 to 45.98)	2699	42.90 (41.67 to 44.14)
Age-adjusted and gender-adjusted prevalence of VI		47.57 (44.33 to 50.83)		36.88 (33.55 to 40.34)		43.31 (40.26 to 46.4)		42.56 (40.52 to 44.63)

VI, vision impairment.

### Risk factors of VI

In the multivariate logistic regression analysis, older age groups had higher odds of having VI. Individuals aged 70–79 years (OR: 1.21; 95% CI 1.07 to 1.36) and those aged ≥80 years (OR: 1.70; 95% CI 1.41 to 2.05) had higher odds than their younger counterparts. Participants with school education (OR: 1.65; 95% CI 1.15 to 2.37) or no formal education (OR: 2.32; 95% CI 1.58 to 3.40) were more likely to have VI than those with higher education. Those reporting diabetes (OR: 0.76; 95% CI 0.66 to 0.88) and hypertension (OR: 0.83; 95% CI 0.74 to 0.93) had lower odds of VI. Individuals who were bedridden/assisted living or had health issues had significantly higher odds (OR: 1.61; 95% CI 1.30 to 1.98) than those who were independent. People who currently smoke (OR: 1.32; 95% CI 1.07 to 1.62), past alcohol consumers (OR: 1.25; 95% CI 1.01 to 1.55) and current alcohol consumers (OR: 1.39; 95% CI 1.12 to 1.73) had higher odds of VI. Non-working (those retired/never employed) individuals had higher odds than those who were currently employed (OR: 1.20; 95% CI 1.05 to 1.37). Residents of the Rayalaseema region had higher odds of VI than those in coastal Andhra Pradesh (OR: 1.38; 95% CI 1.17 to 1.73). The odds of VI were higher for those living in Telangana, the difference was not statistically significant (OR: 1.03; 95% CI 0.86 to 1.25). Although rural residents (p=0.07) and women (p=0.14) had higher odds of VI, these differences were not statistically significant (table 2).

**Table 2:** Association of visual impairment with sociodemographic characteristics and systemic conditions (multiple logistic regression analysis)

	Total in the group (n)	Vision impairment (worse than 6/12 in the better eye) n (row %)	OR (95% CIs)	Statistical significance (p value)
Age group (years)				
60–69	3711	1464 (39.45)	Reference	
70–79	2026	919 (45.36)	1.21 (1.07 to 1.36)	**<0.01**
≥80	554	316 (57.04)	1.70 (1.41 to 2.05)	**<0.01**
Gender				
Male	2865	1202 (41.95)	Reference	
Female	3426	1497 (43.70)	1.11 (0.96 to 1.28)	0.14
Education level				
Higher education	163	42 (25.77)	Reference	
School education	2516	929 (36.92)	1.65 (1.15 to 2.37)	**0.01**
No formal education	3612	1728 (47.84)	2.32 (1.58 to 3.40)	**<0.01**
Diabetes				
No	4928	2226 (45.17)	Reference	
Yes	1363	473 (34.70)	0.76 (0.66 to 0.88)	**<0.01**
Hypertension				
No	3614	1647 (45.57)	Reference	
Yes	2677	1052 (39.30)	0.83 (0.74 to 0.93)	**<0.01**
Mobility status				
Independent	5682	2342 (41.22)	Reference	
Bedridden/assisted living	609	357 (58.62)	1.61 (1.30 to 1.98)	**<0.01**
Smoking status				
Never	4660	1965 (42.17)	Reference	
Previous	891	368 (41.30)	0.98 (0.80 to 1.20)	0.86
Current	740	366 (49.46)	1.32 (1.07 to 1.62)	**0.01**
Alcohol consumption				
Never	4702	1955 (41.58)	Reference	
Previous	762	339 (44.49)	1.25 (1.01 to 1.55)	**0.04**
Current	827	405 (48.97)	1.39 (1.12 to 1.73)	**<0.01**
Employment status				
Working	2463	981 (39.83)	Reference	
Never employed/retired	3828	1718 (44.88)	1.20 (1.05 to 1.37)	**<0.01**
Location				
Urban	1623	617 (38.02)	Reference	
Rural	4668	2082 (44.60)	1.12 (0.99 to 1.27)	0.07
Region				
Coastal Andhra	2093	782 (37.36)	Reference	
Rayalaseema	2059	979 (47.55)	1.38 (1.17 to 1.73)	**<0.01**
Telangana	2139	938 (43.85)	1.03 (0.86 to 1.25)	0.73
Total	6291	**2699** (**42.90**)		

### Causes of VI

Overall, UREs (52.43%; n=1415), cataracts (34.75%; n=938) and posterior capsular opacification (6.41%; n=174) were the leading causes of VI. The proportions of causes varied across regions. UREs were higher in the coastal Andhra region, followed by Telangana and the Rayalaseema region. The proportion of VI due to cataracts was higher in the Rayalaseema region, followed by Telangana and the coastal Andhra region (figure 1).

**Figure 1:**
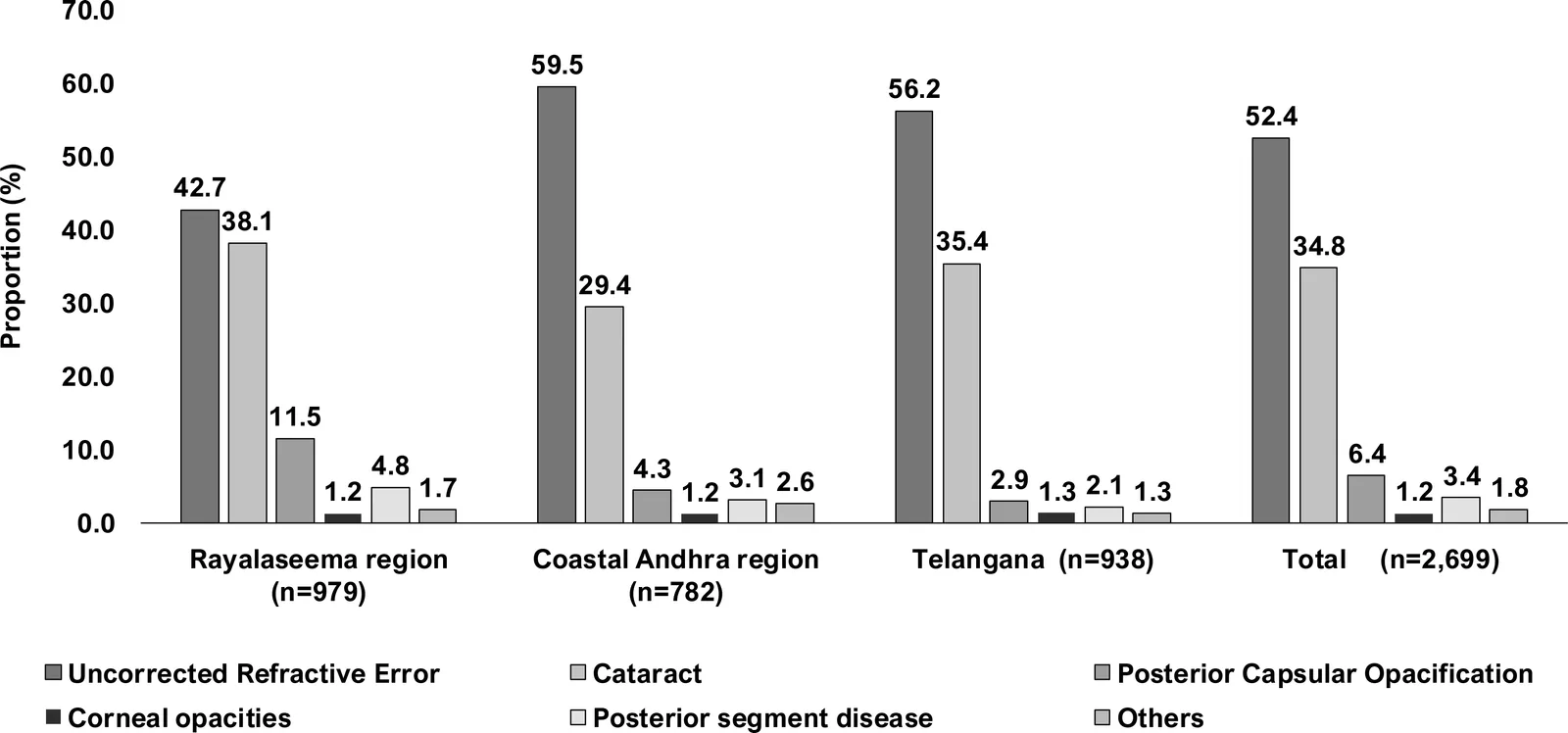
Distribution of causes stratified by region (n=2699).

### Extrapolation of the prevalence estimates to the population

Extrapolation of the data to the older population in two states indicates that more than 2.70 million people, including 1.60 million in Andhra Pradesh and 1.10 million people in Telangana, have avoidable VI. The proportion of older people is projected to increase from approximately 12% in 2023 to 27% in Andhra Pradesh and from 11% in 2023 to 23% in Telangana by 2050. Extrapolating current VI estimates to the growing proportion of older people indicates that the number of people with VI will double to reach 6.46 million by 2050.

Assuming 5 years of disability due to avoidable VI per person, the cumulative YLVDs were 13.45 million person-years in 2023. Even with a conservative assumption of 2 years of disability due to VI, the cumulative YLVDs are a staggering 5.40 million person-years (table 3). Figure 2 shows the cause-specific estimates (figure 2). For 2050, the corresponding YLVDs are estimated at around 32.48 million person-years for 5-year estimates and 13.00 million person-years for more conservative 2-year estimates (table 3).

**Table 3:** Number of people with VI (presenting visual acuity worse than 6/18 in the better eye) and years lived with visual disability in Andhra Pradesh and Telangana

Region	Estimated older population (in millions)	Prevalence of VI (%) in the older population	No. of older persons with VI (≥60 years) (in millions)	Person-years with visual disability (in millions) (5 years)	Person years with visual disability (in millions) (2 years)
Projections for the year 2023					
Andhra Pradesh	6.73	23.77	1.60	8.00	3.20
Telangana	4.31	25.57	1.10	5.50	2.20
Andhra Pradesh and Telangana	**11.04**	**24.38**	**2.70**	**13.45**	**5.40**
Projections for the year 2050					
Andhra Pradesh	15.50	23.77	3.68	18.42	7.37
Telangana	11.00	25.57	2.81	14.06	5.63
Andhra Pradesh and Telangana	**26.50**	**24.38**	**6.49**	**32.48**	**13.00**

VI, vision impairment.

**Figure 2:**
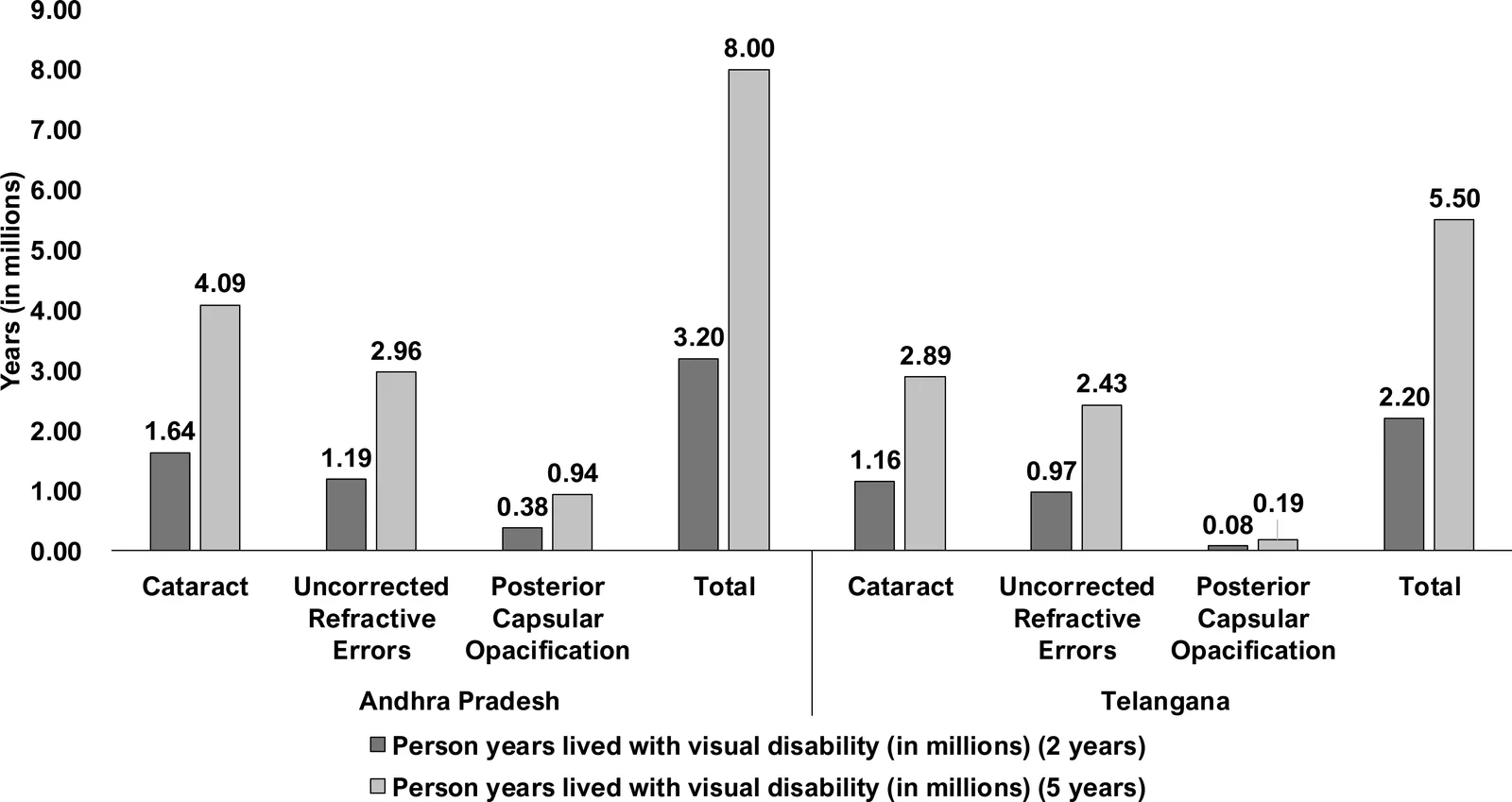
Causes of vision impairment and person-years lived with disability.

## Discussion

Four out of 10 older people in the two states of Andhra Pradesh and Telangana in south India have VI. More than 85% of this VI is due to avoidable causes. Both the prevalence and causes of VI vary across regions in these two states, indicating disparities in service availability and service updates. As in other studies, UREs and cataracts are the leading causes of VI, though the proportions of VI attributable to these conditions vary.^26–28^ Posterior capsular opacification emerged as the third greatest cause of VI. A study from the same region, which included older people living in homes for the aged, also reported a higher proportion of posterior capsular opacification as a cause of VI.^29^

Rapid assessment studies form the cornerstone for estimating the prevalence of VI in India.^30^ Typically, these studies include individuals aged 40 years or older or 50 years or older and thus include a relatively smaller proportion of older people (60 years or older) in the sample.^26 28 30^ Among those aged 40 years and older, the prevalence varied significantly from 6% to 15% in the states of Andhra Pradesh and Telangana using the 6/18 definition.^26 28 31 32^ The national survey (2014–2019) included a district in Rayalaseema, reporting a prevalence of 13.6% (using the <6/18 definition) among those age 50 years and older.^30^ At the national level, the prevalence of VI in the subset of older people was 39.4% compared with 42.9% in the current study using the presenting VA of 6/12 in the better eye definition.^30^

A recent study from Telangana reported on a subsample of older people from four rapid assessment studies and found that a third of them had VI.^33^ However, no similar studies report on the older population in coastal Andhra Pradesh and the Rayalaseema region. One study that included the Akividu region in coastal Andhra Pradesh reported a prevalence of 12.8% among individuals aged 40 years and older and 51% in the subset of older individuals, compared with 42.9% in the current study using <6/18 definition.^28^ The Longitudinal Ageing Study in India (LASI) reported a national prevalence of 33.8% among those aged 45 years and older.^34^ However, this is not comparable due to differences in the age groups included. A few other papers also reported VI from LASI, but the prevalence varied across the studies.^35–38^

As reported in the previous studies, VI was higher in older age groups. Eye conditions such as cataract are more common in older people,^28 30 33^ and they remain a leading cause of VI. Socio-economic reasons such as lack of affordability, accessibility and other family-related issues might have prevented older people from seeking services and so remain visually impaired.^39 40^ In addition, other health priorities could be a potential reason for not accessing services where available as shown in a previous study from the same region.^39^

The national blindness and VI survey indicated a higher prevalence of VI among women; in this study, the prevalence was similar across genders.^30^ Although women had higher odds of VI in the current study, these differences were not statistically significant. The association between VI and lower levels of education is consistent across studies, including this study. The inference is that higher education is associated with better socio-economic status and greater access to resources, enabling this group to use eye care services. Smoking and alcohol consumption are known risk factors for VI. The Andhra Pradesh Eye Disease Study reported a higher prevalence of cataracts among people who smoke and their onset at an early age.^41^ The association between alcohol consumption and VI is rarely reported from India. Evidence from studies conducted in other countries indicated variable associations: some studies reported a positive association similar to the current study, while others found none.^42 43^ Alcohol consumption is likely to result in an earlier onset of cataract leading to VI.^43^ However, information bias may influence the history of smoking and alcohol consumption, as the participants may underreport smoking and alcohol consumption for social reasons, especially among women in India. Both diabetes and hypertension were associated with lower odds of VI. As both these conditions are based on self-report, it is possible that those who reported these two conditions have a higher education level and socio-economic status and have more resources at their disposal to undergo eye examination to address VI.

Demographic shifts and increased longevity are leading to a rise in the proportion of older people who continue to work. In the current study, 40% of respondents were working at the time of data collection. VI is likely to impact their income and work productivity. This can be investiagted in future studies. The causes of VI among older people are largely avoidable with cost-effective interventions such as cataract surgery and spectacles. Given the burden of VI, both in terms of numbers and the large number of YLVDs, the need to prioritise addressing VI in older populations is clear. Enhancing services to provide eye care closer to communities at a cost most can afford is critical. The YLVDs estimates assumed that it takes about 5 years from the onset of cataract to result in at least moderate VI. A more conservative estimate based on 2 years duration is also presented. The Andhra Pradesh Eye Disease Study reported on YLVDs more than two decades ago.^24^ Comparable data on YLVDs in older age groups from other regions in India are lacking, a knowledge gap that future research can address.

India is one of the few countries with a national programme to address VI, which was launched more than five decades ago.^44 45^ It also includes the grant-in-aid programme for cataract and other surgeries conducted in hospitals run by non-government organisations. This initiative has significantly increased the cataract surgical rate (number of surgeries done per million population in a year) and cataract surgical coverage.^46 47^ The recent national survey reported a significant decline in the prevalence of blindness over time; however, it also noted large regional variations.^30^ This underscores the need for regional studies to plan local eye care services, followed by targeted interventions. The vision centres model pioneered by the L V Prasad Eye Institute and implemented by several organisations in India is one effort to bring eye care closer to communities especially for vulnerable groups.^48^ Health and wellness centres are another primary healthcare initiative by the government of India, providing care with a focus on older populations.^49 50^

The last decade witnessed large universal eye health initiatives in both Andhra Pradesh and Telangana.^51^ While the initiatives in Telangana focused on correcting refractive errors, the one in Andhra Pradesh focused on cataract surgery in older adults. Massive efforts were made to provide eye care to a large number of people in a programmatic manner. More such efforts are needed at regular intervals. This becomes even more relevant given a rapidly ageing population. Sustainable, long-term initiatives are needed to address vision loss among older populations.

LEADS is the first comprehensive eye health and ageing study conducted in the older age group in Andhra Pradesh and Telangana (erstwhile undivided state of Andhra Pradesh). This study has several strengths, including the use of a large, population representative sample randomly selected from seven districts; a high response rate; the inclusion of several personal, social and demographic risk factors; and a robust eye-examination protocol. However, a few limitations should be noted, including a higher mean age among those examined than among those not examined. The effect of this higher age may have had minimal influence on the prevalence estimates reported in the study. In conclusion, LEADS provided population-representative estimates of the prevalence and causes of VI in the Andhra Pradesh and Telangana in South India. These estimates can serve as benchmarks for strategic planning and the delivery of eye care services, contributing to the overarching goal of ‘healthy, happy, and active ageing’ in India.

## Data Availability

Data are available upon reasonable request.
